# Placental studies elucidate discrepancies between NIPT showing a structural chromosome aberration and a differently abnormal fetal karyotype

**DOI:** 10.1002/pd.5531

**Published:** 2019-08-13

**Authors:** Diane Van Opstal, Stefanie van Veen, Marieke Joosten, Karin E.M. Diderich, Lutgarde C.P. Govaerts, Joke Polak, Nicole van Koetsveld, Marjan Boter, Attie T.J.I. Go, Dimitri N.M. Papatsonis, Krista Prinsen, Lies H. Hoefsloot, Malgorzata I. Srebniak

**Affiliations:** ^1^ Department of Clinical Genetics Erasmus Medical Center Rotterdam The Netherlands; ^2^ Department of Obstetrics and Fetal Medicine Erasmus Medical Center Rotterdam The Netherlands; ^3^ Department of Obstetrics and Gynecology Amphia Hospital Breda The Netherlands

## Abstract

**Objective:**

Placental cytogenetic studies may reveal the origin of discordant noninvasive prenatal testing (NIPT). We performed placental studies to elucidate discordances between NIPT showing a structural chromosome aberration and the fetus having a different chromosome aberration in three cases.

**Method:**

Diagnostic testing with genomic SNP microarray was performed in three cases with NIPT showing a duplication on 4q (case 1), a terminal deletion of 13q (case 2), and a terminal deletion of 15q (case 3). Placental studies involved SNP array analysis of cytotrophoblast and mesenchymal core of chorionic villi of four placental quadrants. Clinical follow‐up was performed as well.

**Results:**

Amniotic fluid revealed a different structural chromosome aberration than predicted by NIPT: a terminal 2q deletion (case 1), a segmental uniparental isodisomy of 13q (case 2), and a terminal duplication of 15q and of 13q (case 3). Placental studies revealed the aberration detected with NIPT in the cytotrophoblast, whereas the fetal karyotype was confirmed in the placental mesenchymal core.

**Conclusion:**

Our study shows that targeted cytogenetic investigations for confirmation of NIPT showing a microscopically visible structural chromosome aberration should be avoided, since another aberration, even a submicroscopic one or one involving another chromosome, may be present in the fetus.

**What's already known about this topic?**

- Noninvasive prenatal testing (NIPT) investigates cell‐free DNA derived from the cytotrophoblast of chorionic villi.
- NIPT detects placental chromosome aberrations that may be absent in the fetus.
- The majority of discordant NIPT results with a normal fetal chromosome constitution, originates from confined placental mosaicism.

**What does this study add?**

- For confirmation of abnormal genome‐wide NIPT showing a microscopically visible structural chromosome aberration, targeted diagnostic confirmatory testing, only investigating the involved chromosome aberration, should be avoided since the fetal chromosome aberration may involve another chromosome or another structural aberration type. Moreover, no karyotyping, but a SNP array should be the method of first choice, since the fetal chromosome aberration may be submicroscopic whereas NIPT predicts a microscopically visible aberration.

## 1 INTRODUCTION

Noninvasive prenatal testing (NIPT) like other prenatal screening methods has focused on the detection of the most common chromosome aberrations, trisomies 21, 18, and 13, with or without sex‐chromosomal aneuploidies. However, by using genome‐wide sequencing or an array approach, other fetal chromosome aberrations such as other autosomal trisomies as well as structural chromosome aberrations can be detected, as was shown recently.^1^, ^2^, ^3^ The resolution of these genome‐wide approaches is mostly limited to the detection of large, microscopically visible fetal chromosome aberrations of greater than 10 to 15 Mb (eg, subchromosomal aberrations). The detection of specific submicroscopic chromosome aberrations (eg, microdeletions and microduplications, typically less than 5 Mb in size) also have been described, and nowadays, these are sometimes included in commercial NIPT‐kits.

Since the fetal part of the cell‐free DNA is derived from the cytotrophoblast (CTB) of chorionic villi (CV), NIPT in fact detects placental chromosome aberrations. Although the chromosome constitution of placenta and fetus are expected to be the same in most cases, it is known, mainly from CV studies, that discrepancies may occur in 1% to 2% of CVS, at least in a high‐risk population.^4^ Confined placental mosaicism (CPM) is the main reason for discordant NIPT results.^5^ In a previous study, we showed that about 15% of rare autosomal trisomies (RAT, autosomal trisomies different from trisomies of chromosomes 13, 18, and 21) that were detected with NIPT in pregnancies with abnormal first trimester combined (FTC) test results were confirmed in the fetus, but the rest mainly showed to be confined to the placenta.^1^ In contrast, in that same study, it was shown that 50% (six out of 12) of large, microscopically visible (greater than 10 Mb), structural chromosome aberrations that were detected with NIPT, were confirmed in the fetus. This is in line with other papers showing a confirmation rate of 50% to 62%.^2^, ^6^

Follow‐up investigations after abnormal NIPT often are limited to fetal, and depending on the NIPT result, also maternal cytogenetic investigations, and if this reveals normal results, the NIPT is called “false positive” or “discordant.” In most cases, it may then be assumed that the chromosome aberration probably has a placental origin due to the cytotrophoblastic origion of the cfDNA. However, proof is only delivered if placental studies are performed. Since in most cases, an amniocentesis instead of chorionic villus sampling (CVS) is recommended according to a joint European Society of Human Genetics (ESHG)/American Society of Human Genetics (ASHG) position statement,^7^ only postnatal placental studies can proof a placental origin of an aberration detected by NIPT.

In this paper, we present the results of placental follow‐up investigations that were performed in three cases of abnormal NIPT showing a structural chromosome aberration and another abnormal fetal karyotype involving a different structural aberration in an effort to elucidate the observed discrepancies.

## 2 MATERIALS AND METHODS

We present three cases in which NIPT revealed a structural chromosome aberration and in which fetal, placental, and maternal cytogenetic follow‐up investigations during and after pregnancy were performed in order to elucidate the discrepancies that were found between the abnormal NIPT (partial duplication of 4q, a partial deletion of 13q, and a partial deletion of 15q), and differently abnormal fetal karyotype (with respectively a terminal deletion of 2q, a segmental uniparental disomy of 13q and a partial duplication of 13q and 15q).

In all cases, NIPT was performed as part of the Dutch Trident 1 study (Trident = Trial by Dutch laboratories for Evaluation of NIPT). Trident 1 is a nationwide study in which NIPT is offered as an alternative option to invasive testing in patients at elevated risk for trisomy 21, 18, or 13, mostly through abnormal combined test results. A license for the study was granted by the Minister of Health (11016‐118701‐PG). All eight University Medical Centers participate in the study.^1^, ^8^ In our center alone, 2305 samples were processed during the first 3 years (1 April 2014 to 1 April 2017). A total of 12 structural chromosome aberrations were found, of which six were maternal in origin and six had a fetal origin. Of the latter, three are presented in this paper. The method that was used shortly involved genome‐wide shallow massively parallel shotgun sequencing and genome‐wide analysis with WISECONDOR that has a resolution of approximately 15 Mb at a sequencing depth of about 10 to 12 million reads per sample. Sex chromosomes were not analyzed.

Pretest counseling about the different options (invasive testing, NIPT or no testing) was performed by a gynecologist at a University Medical Center for prenatal diagnosis. Pregnant women were informed on the nature of the NIPT test and the possible finding of another chromosome aberration than the one for which they had an increased risk (trisomy 21, 18, or 13). Posttest counseling in case of an abnormal NIPT result was performed by a clinical geneticist.

Follow‐up fetal diagnostic investigations of uncultured amniotic fluid (AF) during pregnancy and of uncultured umbilical cord blood and buccal swab after birth were performed with SNP array (Illumina Infinium_CytoSNP_850K genotyping array). In all cases, karyotyping or fluorescent in situ hybridization (FISH) of AF cell cultures (in situ method) were performed as well. In one case, buccal swab was investigated with FISH instead of SNP array. Cytogenetic investigations of parental blood was performed with the same SNP array or with karyotyping (depending on the chromosome aberration). To identify the parental origin of the segmental uniparental disomy of chromosome 13 in case 2, SNPs were compared between mother and fetus as described previously.^9^

Placental studies after birth involved the analysis of four CV biopsies from four quadrants of the placenta. Both cell layers of CV, the CTB and mesenchymal core (MC), were separated according to standard techniques.^9^, ^10^ After digestion of the MC with collagenase, a part of the cell suspension was cultured according to standard techniques (long‐term cultured villi [LTC‐villi]) and a part was used for DNA isolation. Genomic DNA was also isolated from the CTB; 50 to 100 ng of DNA was hybridized to the Illumina Infinium_CytoSNP_850K genotyping array. For analysis, Genome Studio (Illumina) and different versions of Nexus Copy Number (BioDiscovery, versions 7.0 and higher) were used. In one case, karyotyping of LTC‐villi was performed as well.

We collected clinical outcome data such as birthweight, gestational age, and presence of congenital malformations in the three cases.

## 3 RESULTS

### 3.1 Fetal and parental cytogenetic follow‐up studies

**Figure 1:**
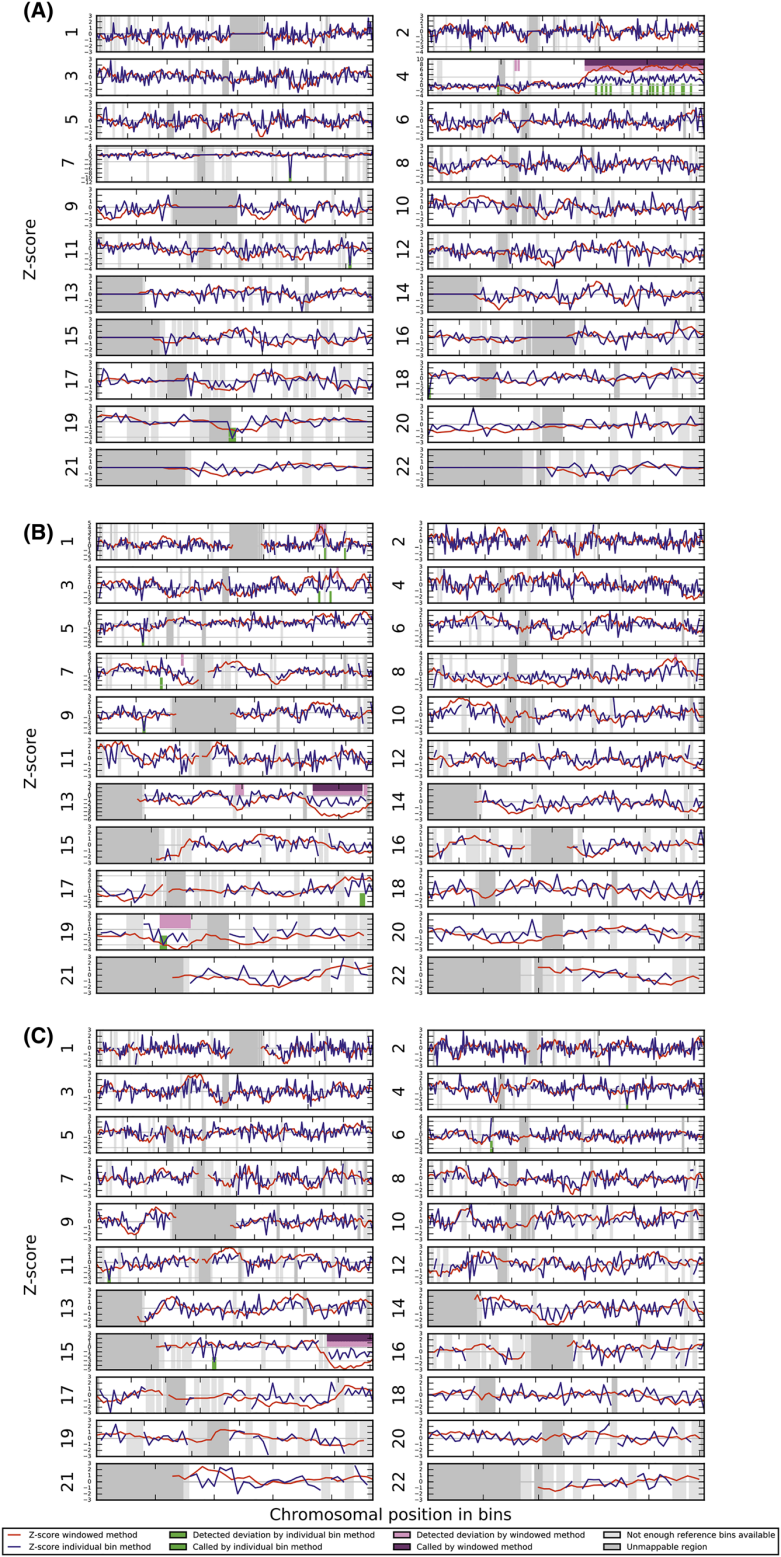
WISECONDOR plots showing the abnormal NIPT results in cases 1, 2, and 3. (A) Case 1 with a duplication of part of 4q—dup(4) (q25q35.2). (B) Case 2 showing a terminal deletion of the long arm of chromosome 13—del(13)(q31). C, Case 3 with a terminal deletion of the long arm of chromosome 15‐ del(15)(q25) [Colour figure can be viewed at http://wileyonlinelibrary.com]

The results of cytogenetic investigations of AF, cord blood, and/or buccal swab after birth and of parental blood for confirmation of an abnormal NIPT result are shown in Table 1. In all three cases, another chromosome aberration than the one predicted by NIPT was found in the fetus:

- In case 1, NIPT detected a duplication on 4q (Figure 1A), while the fetus had a terminal deletion of 2q.
- In case 2 a deletion on 13q as detected with NIPT (Figure 1B) showed to be a segmental uniparental isodisomy (UPiD) of maternal origin of the terminal part of 13q in fetal cells. The PCCA gene in this region was screened for mutations; none were found.
- In case 3 with a deletion of distal 15q in NIPT (Figure 1C), a mosaic duplication of the distal part of the long arms of chromosomes 13 and 15 was found in AF cells.

**Table 1:** Cytogenetic details in the presented cases: abnormal NIPT showing a structural chromosome aberration, prenatal and postnatal follow‐up cytogenetic studies, and clinical outcome

	Indication for Prenatal Testing, US Results and Clinical Outcome	NIPT Result	Fetal Results (Summary)	Prenatal Cytogenetic Results	Results of Placental Studies and Postnatal Cytogenetics
1	aFTS DS 1:167 NT 1.9 maternal age: 38 y Normal US at 16 wk, at time of amniocentesis TOP at 19 6/7 wk Boy, 290 g	dup(4) (q25q35.2)	del(2)(q37.1)	**Amniocentesis** 16 wk: ‐SNP array: de novo loss of 10 Mb in band 2q37.1q37.3 arr[hg19] 2q37.1q37.3(232,717,857‐243,048,760)x1dn ‐Karyotype: 46,XY,del(2)(q37.1)[23] **Parents** Normal karyotypes: 46,XY and 46,XX	**4 placenta biopsies**: **‐CTB of biopsies 1‐4:** Biopsies 1 and 2: chr4:100% dup4q chr2:100% 3 Mb del Biopsy 3: chr4:normal chr2:mos 3 Mb/4 Mb/56 Mb del Biopsy 4: chr4:normal chr2:mos 3 Mb/4 Mb del **‐MC of biopsies 1‐4:** Biopsy 1: chr4:normal chr2:100% 3 Mb del Biopsy 2: chr4: normal chr2: mos 2 Mb/3 Mb/10 Mb del Biopsies 3 and 4: chr4:normal chr2:mos 3 Mb/10 Mb del
2	aFTS DS 1:18, NT 1.9 mm maternal age 35 y Normal US at 18 6/7 wk, 33 4/7 and 36 4/7 wk Healthy girl, born at 38 + 1 w, 3120 g, no congenital malformations. Normal development at 2 y of age.	del(13)(q31)	Segmental matUPiD 13q31.3q34	**Amniocentesis** 20 2/7 wk: ‐SNP array: ~25 Mb ROH on 13q31.3q34 (maternal segmental UPiD) arr[hg19] 13q31.3q34(90252671_115103529)x2 hmz mat ‐FISH on cultured cell clones: normal nuc ish 13q14(PN13x2),13qter(RP1‐1L16x2)[4]. ish 13q14(PN13x2),13qter(RP1‐1L16x2)[28] **Parents** Array normal	**4 placenta biopsies:** **‐CTB of biopsies 1‐4:** Biopsy 1: mos 61.1 Mb del 13q14.3q34 a Biopsy 2: mos 57.2 Mb del 13q21.1q34 a Biopsy 3: mos 27.6 Mb del 13q31.1q34 a and 19.2 Mb mosaic gain in 4p16.3p15.31 Biopsy 4: mos 30 Mb del 13q31.3q34 a **‐MC of biopsies 1‐4:** ~25 Mb ROH on 13q31.3q34 **Cord blood and buccal mucosa:** ~25 Mb ROH on 13q31.3q34 (matUPiD 13q31.3q34) arr[hg19] 13q31.3q34(90252671_115103529)x2 hmz mat
3	aFTS DS 1:63, NT 1.7 mm maternal age 27 y Normal US at 20, 23 6/7, 27 6/7, and 32 wk Male newborn at 38 + 5 wk, birth weight 3338 g induced labor due to mild preeclampsia. Normal development at the age of 2 y	del(15)(q25)	Mosaic gain 15q22.31q26.3 gain and mosaic 13q33.2q34 gain	**Amniocentesis** at 16 4/7 wk ‐SNP array: mos ~36 Mb gain of 15q22.31q26.3 and ~10 Mb gain of 13q33.2q34 (~5% in uncult cells and ~5%‐10% in cell cultures) arr[hg19] 15q22.31qter (66,612,725‐102,461,162)x2~3, 13q33.2qter(105,015,223‐115,103,529)x2~3 dn ‐Karyotyping: 46,XY,add(13)[4]/46,XY[12] ‐FISH**:** 15qtelomere (RP1‐154P1) and 15q26.2 (RP11‐784A9 and RP11‐337 N12): add(13) = t(13;15)(q34;q22.31) in 8/26 cell clones (31%) **Parents** Array normal	**4 placenta biopsies** **‐CTB of biopsies 1‐4:** chr15: mos 17 Mb del q25.3q26.3/62.5 Mb dup q11.2‐q25.3 chr13: normal **‐MC of biopsies 1‐4:** chr15: mos ~36 Mb gain of 15q22.31q26.3 chr13: mos ~10 Mb gain of 13q33.2q34 Karyotyping of MC of biopsy 1: 46,XY,der(13)(t(13;15) (q34;q22.31))[11]/46,XY[2] **Cord blood.** Array normal **Buccal mucosa** FISH: 2.5% 3 signals with 15qtel probe (10/400 nuclei)

*Note*. The chromosome aberration as found with NIPT (in red) was confirmed in the cytotrophoblast of placental chorionic villi, whereas the fetal chromosome aberration, as found in amniotic fluid (in green), was confirmed in mesenchymal core of placental chorionic villi.

Abbreviations: aFTS, abnormal first trimester screening; cell cult, cultured cells; chr, chromosome; CTB, cytotrophoblast; del: deletion; dup, duplication; matUPiD, maternal uniparental isodisomy; MC, mesenchymal core; TOP, termination of pregnancy; uncult, uncultured; US, ultrasound; −, not available.

a The other cell line is the one with approximately 25‐Mb ROH on 13q31.3q34 that was also detected in MC, AF, cord blood, and buccal mucosa.

### 3.2 Placental cytogenetic confirmatory testing

**Figure 2:**
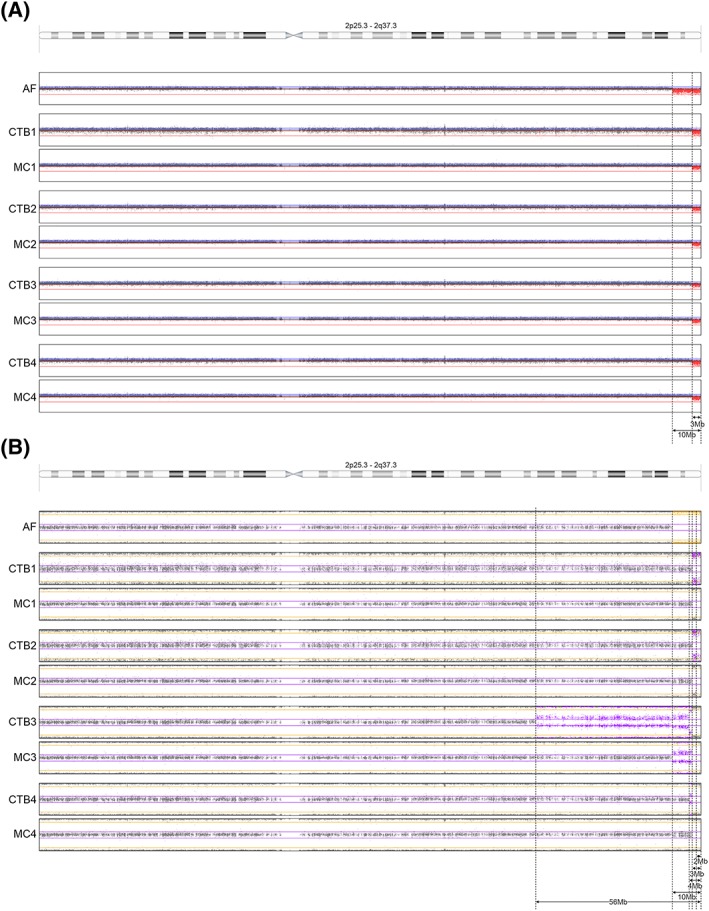
LogR (A) and B‐allele frequency (BAF) (B) plots of chromosome 2 in different tissues of case 1: AF, amniotic fluid; CTB1, 2, 3, and 4, cytotrophoblast of placental biopsies 1, 2, 3, and 4; MC1, 2, 3, and 4, mesenchymal core of placental biopsies 1, 2, 3, and 4. (A) Ideogram of chromosome 2 and LogR plots: the LogR shows a 10‐Mb deletion in AF while the CTB and MC of all placental biopsies show a much smaller deletion of 3 Mb. (B) Ideogram of chromosome 2 and BAF profiles showing a 100% deletion of 10 Mb in AF and a 100% 3 Mb deletion in CTB1, MC1, and CTB2 confirming the LogR. It should be noted that in both CTB biopsies, a 5% to 10% maternal cell contamination is visible so that the BAF profiles resemble that of an approximately 90% to 95% mosaic. Mosaicism of different lengths deletions (of 2, 3, 4, 10, and 56 Mb) is seen in the other biopsies, with the 3‐Mb deletion being the predominant cell line. MC2 shows mosaicism of a 2‐, 3‐, and 10‐Mb deletion. Only knowledge of the presence of a 10‐Mb deletion cell line in the conceptus reveals its presence in MC2. CTB3 shows a mosaic 3‐, 4‐, and 56‐Mb deletion. MC3 shows a mosaic 3‐ and 10‐Mb deletion. CTB4 shows mosaicism of a 3‐ and a 4‐Mb deletion. MC4 shows a mosaic 3‐ and 10‐Mb deletion [Colour figure can be viewed at http://wileyonlinelibrary.com]

**Figure 3:**
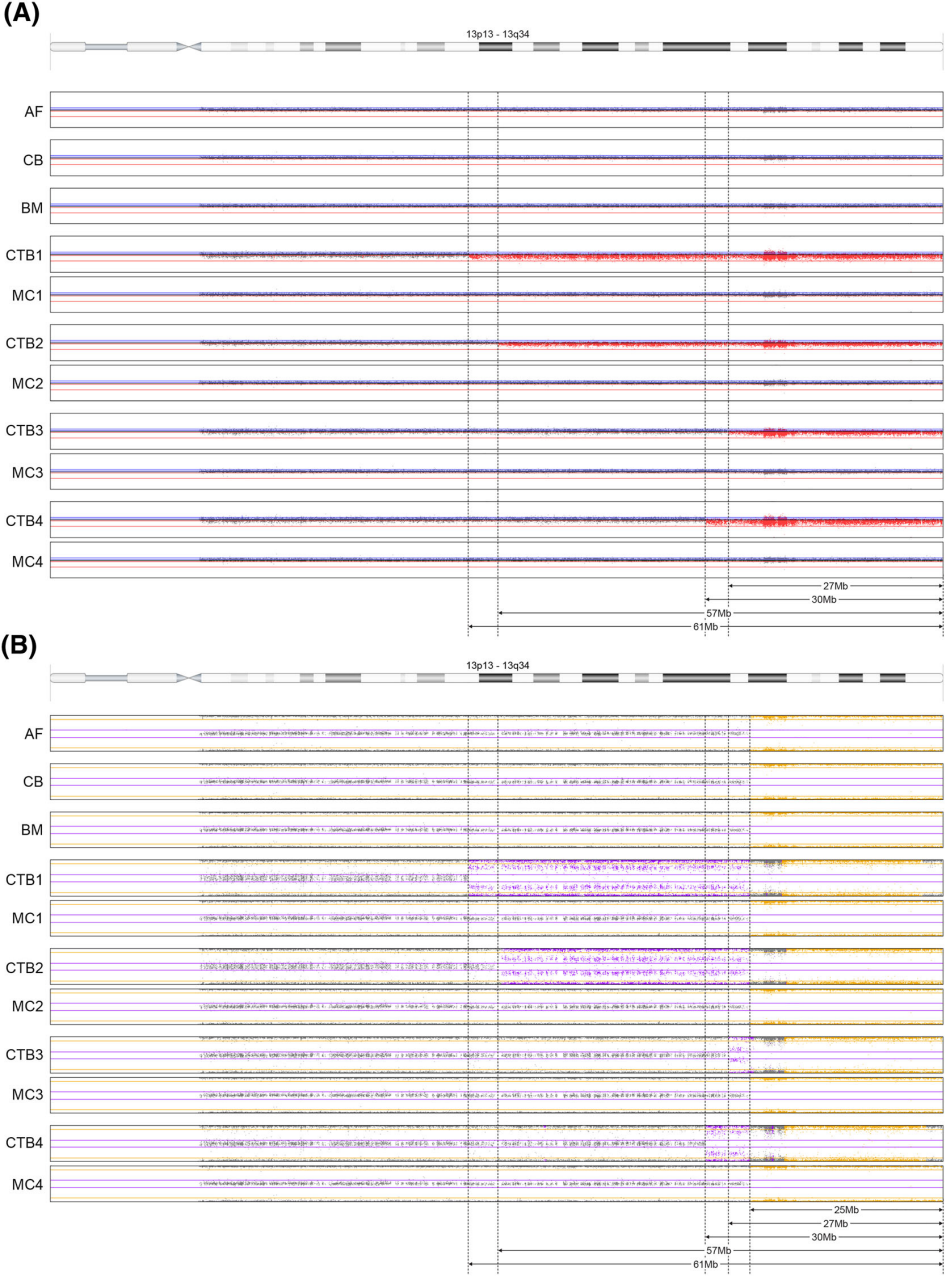
LogR (A) and B‐allele frequency (BAF) (B) plots of chromosome 13 in different tissues of case 2: AF, amniotic fluid; BM, buccal mucosa; CB, cord blood; CTB1, 2, 3, and 4, cytotrophoblast of placental biopsies 1, 2, 3, and 4; MC1, 2, 3, and 4, mesenchymal core of placental biopsies 1, 2, 3, and 4. (A) Ideogram of chromosome 13 and LogR plots in different tissues: AF, CB, BM, and MC1‐4: the LogR shows a normal result. CTB1‐4 show a 61‐, 57‐, 27‐, and 30‐Mb deletion, respectively. (B) Ideogram of chromosome 13 and BAF profiles in different tissues: AF, CB, BM, and MC1‐4: all BAF plots show a 25‐Mb ROH, fitting a segmental UPiD13. CTB1‐4 all show mosaicism for a 61‐, 57‐, 27‐, and 30‐Mb deletion, respectively, with 25‐Mb segmental UPiD13 (in 13q31.3q34) in the normal cell line [Colour figure can be viewed at http://wileyonlinelibrary.com]

In an effort to elucidate the discordances between NIPT and prenatal diagnosis, placental studies were performed. In all three cases, analysis of placental CTB revealed the chromosome aberration that was found with NIPT, while the chromosome constitution of the MC was representative for that of the fetus:

- Case 1:
  ○ CTB: The duplication on chromosome 4q was indeed detected in the CTB of two of four placental biopsies, which confirmed the NIPT result. Moreover, a mosaic of different length deletions of chromosome 2q (of 3, 4, and 56 Mb) was found (Figure 2).
  ○ MC: The 10‐Mb 2q deletion that was seen in the fetus, and which was absent in the CTB, was present in the MC of biopsies 2, 3, and 4, although at a low level as based on the B‐allele frequency (BAF) profiles. The most prominent abnormal cell line was a 3 Mb deletion (Figure 2).
- Case 2:
  ○ CTB: A mosaic terminal deletion of 13q was detected in the CTB of all four placental biopsies. However, in each biopsy, the deletion had a different length, ranging from 27.6 up to 61.1 Mb, with the other cell line showing a copy number neutral region of homozygosity (ROH) of 25 Mb on 13q31.3q34. The latter was also found in AF, cord blood, and buccal mucosa as well as in all four MCs of placental villi (Figure 3). Moreover, analysis of biopsy 3 also revealed a 19.2‐Mb mosaic gain in 4p16.3p15.31.
  ○ MC: the MC of all four biopsies showed a 25‐Mb ROH that was also seen in the fetus (Figure 3).
- Case 3:
  ○ CTB: The terminal deletion on chromosome 15 was confirmed in the CTB of all four placental biopsies showing a mosaic 17 Mb deletion. In addition, a low mosaic duplication of 62.5 Mb, ranging from 15q11.2 to 15q25.3, was present as well in all biopsies
  ○ MC: In the MC of the four biopsies, the same chromosome constitution as in AF with a mosaic duplication of the terminal 36 Mb of 15q and the terminal 10 Mb of 13q was found, but at a much higher level of between 60% and 80% depending on the biopsy.

### 3.3 Clinical outcome

Clinical outcome data are shown in Table 1. In case 1 with a terminal 2q deletion, causing the well described 2q terminal deletion syndrome, the pregnancy was terminated. In cases 2 and 3, with respectively a segmental maternal UPiD13 (case 2) and low level mosaicism of partial trisomies 13 and 15 (case 3), (apparently) healthy children were born at an appropriate gestational age, both showing normal development at the age of 2 years.

## 4 DISCUSSION

We present three cases in which extensive placental cytogenetic studies revealed an explanation for the discordances between NIPT showing a structural chromosome aberration and the abnormal fetal karyotype involving a different chromosome aberration: In all three cases, the CTB of placental CV demonstrated the chromosome aberration found with NIPT, while the MC of the placental CV showed the fetal chromosome aberration. This again proves the cytotrophoblastic origin of the cf fetal DNA..^11^, ^12^ Moreover, it again demonstrates that the MC is better representative for the fetal chromosome constitution due to the same embryonic origin.^13^

In all three cases, chromosomal mosaicism involving multiple abnormal cell lines that originated during early embryogenesis with unequal distribution of abnormal cells over the different compartments of CV and fetus explained the discordances. On a total of 2305 blood samples from high‐risk pregnancies (cases with abnormal FTC test results) that we investigated during the first 3 years of trident 1 in our center, 12 structural chromosome aberrations were found with NIPT of which six were of maternal origin and six were fetal (unpublished data from Erasmus MC). In half of these six fetal cases (which are the three cases presented in this paper), we observed this phenomenon of discordancy between NIPT results and fetal chromosome constitution, while in the other three cases, fetal karyotype and NIPT result were concordant. So this does not seem to be an uncommon phenomenon in case NIPT shows a subchromosomal aberration. The use of an SNP array, which is very sensitive for detection of low‐level mosaicism and other submicroscopic aberrations (eg, uniparental disomy (UPD) and microdeletion/duplications) may contribute to this high percentage of discrepancy. Nevertheless, our results warrant the use of a genome‐wide test over targeted testing for confirmatory cytogenetic investigations of CV or AF. For instance, FISH with a 4q‐probe in case 1 would never have revealed the fetal 2q‐terminal deletion. Moreover, we believe that SNP array instead of karyotyping or array CGH is the appropriate genome‐wide test since the segmental UPD in case 2, would not be detected with the latter techniques, and would potentially be of major clinical relevance if an imprinted chromosome was involved. Apart from an increased risk for a recessive disease involving a gene in the ROH, there was no clinical effect to be expected in this particular case of segmental UPD13. Also in case 1, the terminal 10‐Mb deletion of 2q may be missed with karyotyping, although its resolution is 5–10 Mb, but this will highly depend on the quality of the chromosome preparations.

In all three placentas, complex mosaicism was found. In the cases involving a deletion on chromosomes 2 (in the fetus and MC) and 13 (in all CTBs of the placenta), deletions of four different lengths were found in the four placenta biopsies. Likewise, in the case with a terminal duplication of 15q (in the fetus), a more proximal duplication was found in the CTBs. These cytogenetic results probably demonstrate the mitotic chromosome instability seen in cleavage stage embryos.^14^ It is striking that in the placenta of case 1 (deletion of chromosome 2), most biopsies revealed mosaicism of different length deletions whereas in the placenta of case 2 (deletion of chromosome 13), each biopsy showed one deletion but all of a different length. Perhaps, this demonstrates the instability of the chromosome 13 deletion during early embryogenesis, which became stable during placental development in case 2, whereas the reverse occurred in case 1, although we admit that this is very speculative. Moreover, in one of the three placentas (case 2), cytogenetic studies revealed an extra chromosome aberration (a mosaic duplication of the short arm of chromosome 4) in one CTB biopsy that was not seen prenatally (with NIPT or invasive testing). This phenomenon of extra chromosome anomalies in the placenta was recently described in 2/10 placentas that were investigated in order to confirm abnormal NIPT involving a numerical chromosome aberration.^9^

Unfortunately, it could not be investigated whether there is a cytogenetic association between the duplication of chromosome 4q that was detected with NIPT and the terminal deletion of 2q (case 1). The 4q duplication was only present in the CTB of two biopsies, and these samples were not cultured but used entirely for DNA isolation for SNP array (therefore, no chromosome preparations were available). However, it is possible that the 4q was “captured” by the 2q terminal deletion for telomere stabilization, at least in one of the early embryonic cells that was allocated to the CTB. It was recently shown that distinct stabilizing events, telomere healing (eg, de novo telomere addition mediated by telomerase) and telomere capture from a different chromosome, resulting in a derivative chromosome, of the same terminal deletion can occur in different early embryonic cells.^15^, ^16^ The mosaic karyotype observed in case 2 with terminal deletions of different lengths of chromosome 13q and with a segmental UPD of the distal 25 Mb on 13q is another example of postzygotic telomere stabilization through telomere capture as well as telomere healing in different embryonic cells.^15^, ^16^ Telomere capture here involved the acquisition of a new telomere sequence from a chromatid from the normal homologue, resulting in a maternal segmental UPiD13. Since not a deletion, but only the segmental UPD was present in MC as well as in the fetus (AF, cord blood, and buccal swab), and since both cell lines were present in the CTB, an early repair in one of the first cleavage divisions (before differentiation in trophectoderm and inner cell mass) of a meiotic terminal deletion of approximately 25 Mb is most likely. Subsequently, only cells with the segmental UPiD were allocated to the inner cell mass giving rise to 100% segmental UPiD13 in MC and fetus. The approximately 25‐Mb deletion cell line persisted in the trophoblast and gave rise to larger deletions of approximately 27, 30, 57, and 61 Mb during further development.

The 2q deletion in case 1 could not be detected with our NIPT test since in the CTB's of all CV biopsies, a much smaller deletion of 3 or 4 Mb was present in the majority of cells. Only low‐level mosaicism of a larger 56‐Mb deletion was present in one of the biopsies, which probably did not result in a sufficient contribution to the cfDNA pool in maternal plasma, so that it remained undetected with our NIPT approach, characterized by a resolution of 10 to 15 Mb.^17^

In the era of NIPT, which investigates cfDNA that originates from the CTB of CV, placental cytogenetic investigations are in the spotlight again. Placental studies used to be frequently performed after the introduction of CVS in the 80s of last century,^18^ and that led to an exponential increase of our knowledge of CPM and its clinical relevance. In our opinion, placental studies for confirmation of an abnormal NIPT result are important for several reasons:

- If a placental origin is proven, another source for the abnormal cfDNA, such as a maternal tumor, can be excluded.
- For reassurance of the parents that may be anxious after an abnormal NIPT despite normal cytogenetic results from AF.
- For increased knowledge of the origin of abnormal NIPT and its associated clinical impact.
- For better interpretation of abnormal results and therefore improved pre‐as well as posttest counseling.
- Finally, for better insight into the true performance of the NIPT test, probably reaching a positive predictive value of 100% if all possible sources of the cfDNA would be investigated.

The main conclusion of this study is that the use of targeted cytogenetic investigations for confirmatory diagnostic testing of NIPT showing a structural chromosome aberration should be avoided since another chromosome aberration, even involving another chromosome, may be present in the fetus. Moreover, also the use of karyotyping should be discouraged and replaced by preferably SNP array since a submicroscopic structural aberration or segmental UPD may be present in the fetus even though the NIPT predicts a microscopically visible chromosome aberration.

## CONFLICT OF INTEREST

None declared.

## Data Availability

The data that support the findings of this study are available from the corresponding author upon reasonable request.
